# 3D reconstruction of lower anterior teeth from CBCT images: automatic segmentation with manual refinements

**DOI:** 10.1590/2177-6709.28.3.e232249.oar

**Published:** 2023-07-17

**Authors:** Gaston Federico Coutsiers MORELL, Kevin CHEN, Carlos FLORES-MIR

**Affiliations:** 1 Private practice (Montreal, Quebec, Canada).; 2 Private practice (Edmonton, Alberta, Canada).; 3 University of Alberta, College of Health Sciences, Faculty of Medicine and Dentistry (Edmonton, Alberta, Canada).

**Keywords:** Root resorption, CBCT, Tooth volume, Root volume, Orthodontics

## Abstract

**Objective:**

To develop a well-detailed and reproducible tooth segmentation method, when quantifying tooth volumetric measurements is needed.

**Material and Methods:**

This was an *in vitro* study in which lower incisors and canines of five patients were 3D reconstructed by means of an automatic segmentation with manual refinements process. All the images were obtained using a 0.3-mm voxel size CBCT imaging. The software utilized was the ITK-SNAP®. The primary outcomes were the intra-rater and inter-rater reliabilities and the respective measurement errors.

**Results:**

The intra-rater reliability was excellent, with a mean measurement error of 4.16%. The inter-rater reliability was good, with a mean measurement error of 7.11%. Accuracy assessment was not possible, as the assessed teeth were not extracted.

**Conclusions:**

Although the described method is reliable, tooth volumetric error measurements may become significant, depending on the assessed situation.

## RESUMO

**Objetivo:**

Desenvolver um método de segmentação dentária bem detalhado e reprodutível, para quando for necessário quantificar as medidas volumétricas dos dentes.

**Material e Métodos:**

Esse foi um estudo *in vitro* no qual incisivos inferiores e caninos de cinco pacientes foram reconstruídos em 3D por meio de um processo de segmentação automática com refinamentos manuais. Todas as imagens de TCFC foram obtidas usando tamanho de *voxel* de 0,3 mm. O *software* utilizado foi o ITK-SNAP®. Os resultados primários foram as confiabilidades intra-avaliadores e interavaliadores e os respectivos erros de medição.

**Resultados:**

A confiabilidade intra-avaliador foi excelente, com erro médio de medição de 4,16%. A confiabilidade interavaliadores foi boa, com erro médio de medição de 7,11%. A avaliação da precisão não foi possível, pois os dentes avaliados não foram extraídos.

**Conclusões:**

Embora o método descrito seja confiável, os erros nas medições volumétricas dos dentes podem se tornar significativos, dependendo da situação avaliada.

## INTRODUCTION

Traditionally, external root resorption (ERR) has been quantified by measuring the length of the root or the entire tooth using two-dimensional (2D) imaging, with the help of panoramic, periapical (PA) and/or lateral cephalogram radiographs. The main problem with this approach is that ERR is a three-dimensional (3D) effect on a 3D structure. Therefore, 2D assessment is expected to be incomplete and inaccurate^1^ and might lead to wrong management decisions.^1^

Magnification errors^2^ and the high subjectivity in the assessment^3^ are sources of inaccuracy from 2D radiograph reconstructions, when compared to CBCT reconstructions. Specifically, magnification is inexistent in CBCT images because their depiction is 1:1, compared to the actual object dimensions. Other problems with 2D radiograph reconstructions are the lack of focus on specific structures and the overlap of different anatomic structures.^4^ These issues are inexistent for CBCT imaging due to their image acquisition characteristics. For all these reasons, a more accurate ERR measurement is expected using CBCT imaging, compared to conventional 2D imaging.

One previous study assessed the accuracy of digital periapical radiographs and CBCT reconstructions when used to diagnose natural and simulated ERR lesions.^5^ The reference standard used was micro-computed tomography (micro-CT). The authors concluded that CBCT reconstructions were the best currently available method to detect ERR, compared to periapical radiographs.

Several specific imaging processing methods to obtain volumetric teeth measurements from a CBCT file have been developed.^6^ ^-^ ^8^ These methods were not necessarily user-friendly for the clinician or the researcher. Fine-tuning 3D imaging processing techniques to assess and quantify ERR are still needed. Thus, the present study proposes a well-detailed and reproducible tooth segmentation method, when quantifying tooth volumetric measurements is required. 

## MATERIAL AND METHODS

### SOFTWARE SELECTION

To bridge the gap between methodological advances and clinical routine, ITK-SNAP^®^ was developed as an open-source, intended to make level set segmentation from CBCT imaging straightforwardly accessible to a wide range of users, including those with little or no mathematical expertise. ITK-SNAP^®^ is also a highly reliable and efficient alternative to manual tracing.^9^

### DESCRIPTION OF THE DEVELOPED 3D RECONSTRUCTION TECHNIQUE

The type of reconstruction performed was an automatic segmentation with manual refinements. The first step of this method consists of constructing 3D volumetric label maps of the teeth, accomplished by the software. The second step consists of manual refinements of the initial automatic segmentations. The contours of each tooth had to be refined layer by layer in each of the three planes of the space. The third step allows segmenting each tooth to assign it to a different layer. The fourth step is a minor refinement of the areas where the teeth were separated. The fifth and last step was to collect the primary dependent variable (volume), to compare all the measurements. An example of a final result of the 3D reconstruction can be observed in Figure 1. More details of the segmentation process are described in Appendix 1.

**Figure 1:**
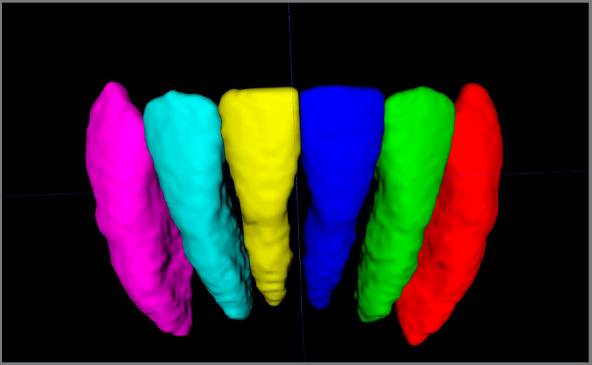
3D volumetric reconstruction of the previously segmented tooth.

### STUDY POPULATION AND DATA COLLECTION

CBCTs were collected from two-time points, before and after the completion of orthodontic treatment, from five randomly selected patients that were participants in a randomized control trial (10% of a total of 44 available participants). The convenience sample size was set at 10% of the total available sample. Ten images were deemed appropriate to develop and test the proposed methodology. All six lower anterior teeth were measured following the previously described technique. The selected teeth were the lower incisors and canines of these patients. Three consecutive measurements of the same six teeth were obtained by the principal investigator (GFCM). Those measurements were taken one week apart from each other. Another investigator, with similar experience and calibration as the principal investigator (KC), took only one set of measurements. All consecutive measurements can be found in Table 1. For the inter-rater reliability analysis, KC’s set of measurements was compared to GFCM’s second set of measurements.

**Table 1:** Consecutive volumetric measurements of each tooth and each patient performed by both researchers, KC and GFCM.

Patient number	Tooth number	GFCM 1^st^ set of measurements	GFCM 2^nd^ set measurements	GFCM 3^rd^ set of measurements	KC unique set of measurements
1	33	393.9	399.1	383.2	358.4
	32	267.5	274	256.7	268.1
	31	232.6	235.6	222.6	212.7
	41	231.6	234.1	221.9	207
	42	266	269.7	256	248.3
	43	409.8	410.4	397.6	373.3
2	33	603.5	572	586.4	497.2
	32	337.3	317.8	331.6	294.7
	31	290.2	286.1	296.9	263.7
	41	284.8	270.1	279.1	273
	42	316.8	298	308.9	279.1
	43	566.5	528.2	536.4	467.3
3	33	667.9	626.8	648.7	670.9
	32	306.6	300.5	313.1	334.9
	31	225.1	224.3	234.5	230
	41	253.7	240.6	255.2	243
	42	319.5	303	320.7	316.1
	43	677.5	624.4	638.3	678.5
4	33	572.8	537.9	566.1	573.2
	32	389.9	348.3	373.2	375.7
	31	334.9	296.9	319	313.9
	41	324.6	286	303.7	305.9
	42	367.7	326.6	352.8	351.5
	43	564.3	519.1	545.6	542.7
5	33	450.6	449	468.8	472.1
	32	256.3	261.4	270.8	279.8
	31	219.8	225	236.2	243.9
	41	227.6	231.8	238.8	246.8
	42	268.2	271.2	273.3	300.5
	43	477.6	473	487.8	484.6

### CBCT CHARACTERISTICS

The dimensions of the full FOV were 16 cm (w) x customized height up to 13 cm. Images included from the roof of orbits to the inferior border of the mandible, around the level of cervical vertebra (C4). FOV was reset in large patients with large mandibular angle/plane with a 16 or 23 cm (w) x up to 17 cm (h) from the level of frontal bone / frontal sinus superiorly to the inferior border of mandible/level of C3-C5. A medium-low resolution was used (0.3 mm voxel and 4.8 seconds). All the CBCT acquisitions were made with the same X-ray machine in the same facility.

## STATISTICAL ANALYSES

The first step was to describe the magnitude of the correlation. The Confidence Intervals (CI) were analyzed. Using the software SPSS^®^ (IBM, Armonk, NY, USA), Intraclass Correlation Coefficient (ICC) tests were performed. A significance level of α=0.05 was chosen. ICC values less than 0.5, between 0.5 and 0.75, between 0.75 and 0.9, and greater than 0.90 indicate poor, moderate, good, and excellent reliability, respectively.^10^ The measurement error was calculated by means of the percentage of variation between measurements regarding the total. 

## RESULTS

### INTRA-RATER RELIABILITY RESULTS

The null hypothesis was rejected for all the teeth (*p* < 0.001). The ICC for all the measurements was >0.910, with an average ICC of 0.94 (Table 2). Therefore, this method displays excellent reliability under the stated conditions.^10^ However, ideally, the limits of the CIs of every tooth measured should be above 0.75 to be good.^10^ This was not the case for the lower boundaries of the CIs of teeth #32, #31, #41, #42, and #43. The average measurement error for all the teeth together was 4.16% (Table 2).

**Table 2:** Intraclass Correlation Coefficients (ICC) values, *p*-values, Confidence Intervals (CI) and measurement errors for the intra-rater reliability analysis

Tooth number	P-value	ICC value	CI Lower limit	CI Upper limit	Measurement error (%)
33	<0.05	0.90	0.27	0.99	8.58
32	<0.05	0.82	0.15	0.98	6.98
31	<0.05	0.87	0.13	0.99	6.90
41	<0.05	0.86	0.10	0.98	5.47
42	0.53	0.75	-0.20	0.97	7.33
43	<0.05	0.91	0.31	0.99	7.38
Average	0.53	0.85	0.13	0.98	7.11

### INTER-RATER RELIABILITY RESULTS

The null hypothesis was rejected (*p*<0.05) for all the teeth except tooth #42. The ICC values reflected good reliability for all the teeth except for tooth #43, indicating excellent reliability. The average ICC was 0.85 (Table 3). Again, ideally, the limits of the CIs of every tooth measured should be, at least, above 0.75 to be good.^10^ The average measurement error for all teeth together was 7.11% (Table 3). Table 4 presents a summary of all the reliability results.

**Table 3:** Intraclass Correlation Coefficients (ICC) values, *p*-values, Confidence Intervals (CI) and measurement errors for the inter-rater reliability analysis.

Tooth number	P-value	ICC value	CI Lower limit	CI Upper limit	Measurement error (%)
33	<0.001	0.99	0.93	1.00	3.51
32	<0.001	0.94	0.73	0.99	4.44
31	<0.001	0.94	0.74	0.99	4.41
41	<0.001	0.91	0.64	0.99	4.52
42	<0.001	0.92	0.65	0.99	4.08
43	<0.001	0.98	0.89	1.00	3.98
Average	<0.001	0.94	0.76	0.99	4.16

**Table 4:** Summary of the results of all inter-rater and intra-rater reliability analyses

Technique	Inter-/Intra-rater	Reliability	Measurement error
Volume	Intra-	Excellent	4.16%
	Inter-	Good	7.11%

## DISCUSSION

### SUMMARY

In the literature, other similar studies have reached reliability values close to the current ones for both intra- and inter-rater tests. The same can be noted about the measurement error, although it is less commonly reported. Details about the contrast, the threshold, and the smoothing adjustments are important due to their influence on the segmentation process. Including the crown and the pulp in the segmentation has logical reasons, discussed as well. Lastly, some limitations are inherent to the CBCT characteristics, and a comparison to a gold standard was not a viable option in this study. The technique developed seems reliable and can be applied when volumetric tooth data is needed.

### RELIABILITY VALUES COMPARED TO OTHER RELATED STUDIES

Regarding the intra-rater reliability, the average ICC value found in this study was 0.94, which corresponds to an excellent level. After segmenting twenty volumes from CBCT 200µm and CBCT 300µm, Maret et al.^11^ found ICCs of 0.998 and 0.999, slightly higher than those obtained in this study and still within the range of excellence. In a study developed by Puttaravuttiporn et al.,^12^ in which upper incisors were segmented, the ICC for intra-rater reliability of tooth volume was >0.90, again similar to the presented results. Therefore, it can be considered that values obtained for intra-rater reliability are within the range of previous related studies.

In an *in-vitro* study comparing laser scans to CBCT scans,^13^ the inter-rater reliability measurements were perfect (ICC=1). In the same range, after segmenting twenty volumes from two CBCT scans with different voxel sizes, CBCT 200µm and CBCT 300µm, Maret et al.^11^ found inter-rater reliability ICCs values of 0.999 and 0.988. In another similar study, Ahlbrecht et al.^14^ segmented maxillary incisors, and they found that the inter-rater reliability for the volume of the repeated models yielded an ICC of 0.98. However, lower values were found in another study, including segmentations from CBCTs. For the inter-rater reliability of that study, the ICC obtained by Liu et al.^15^ was 0.86. This last value is close to the one obtained by the authors of the present study. Consequently, it could be argued that the current inter-rater reliability values are within reasonable reach, but in the lower end of previous similar approaches.

In summary, the intra-rater reliability is excellent and similar to the one reported in previous studies, while the inter-rater reliability is in the lower range of the previously reported values.

### MEASUREMENT ERRORS COMPARED TO OTHER RELATED STUDIES

Interestingly, measurement errors are not commonly reported in related studies. It seems the focus is on reliability only. The measurement error is the variability that indicates that changes around its value or less in the tooth volume could be explained by either measurement error or an actual volume change. If the measurement error is resultant from one of the time points, a more significant measurement error is logically yielded when the difference between T0 and T1 is calculated. The reason is that the measurement errors of the two-time points cumulate.

In an *in-vivo* study conducted by Liu et al.,^15^ the validity of the tooth volume determinations from CBCTs was explored. The raw measurements were published, and even if the authors did not calculate the measurement error, it was calculated by the primary author of the current study based on the available data. The resulting measurement error was 8.24% for the inter-rater analysis, close and slightly higher than the value yielded from the current study (7.11%).

### THRESHOLD AND CONTRAST EMPLOYED IN THE SEGMENTATION PROCESSES

In this method, the upper threshold is placed to the maximum. In contrast, the lower threshold is individually adapted for every patient and every time point. By arbitrary agreement between the researchers involved in this study, KC and GFCM, the lower threshold range was 500 to 1500. What is reported in the literature is that thresholds were set at 56 to 3071 Hounsfield units (HU), minimum and maximum, respectively. If the HU threshold is set too high, the tooth contour cannot be obtained entirely, and tooth volume tends to be smaller. If the HU threshold is set too low, the surrounding tissues will significantly impact the tooth contour, and the tooth volume tends to be larger.^13^ Likely, standardized ranges have not been developed due to the variability between CBCT machines. 

The problem with the previously mentioned variability is that it can also be found even within the same machine in different acquisitions and among patients and bone and teeth densities. Indeed, Liu et al.^15^ concluded that visual adjustments of threshold parameters resulted in different threshold levels for different teeth in the same DICOM data sets and between different data sets. Therefore, the individual adaptation of the threshold within arbitrary limits is the best solution currently supported. Furthermore, using a global threshold for each segmentation is not supported by Liu et al.^15^

Also, teeth density is very different from the crown to the apex. If a single parameter was applied for the segmentation of the whole tooth, it might not be possible to visualize the crown and the root apex simultaneously.

As described in Appendix 1, the contrast selection was similar to the threshold selection: a subjective visual assessment of the structures to be segmented. The intention was to see better and segment the regions of interest. The contrast selection, as well as the threshold selection, may have affected the final results. Probably, despite the good reliability results obtained for both techniques developed, a completely different study would be necessary to be able to quantify the influence of contrast and threshold selection in the outcome precisely.

### SMOOTHING OPTION PROVIDED BY THE SOFTWARE

As explained in the step-by-step segmentation process in Appendix 1, the smoothing options offered by ITK-SNAP^®^ were turned off. Smoothing enhances the visual appearance of the 3D model. Nonetheless, it can also reduce the volume from three to twelve percent, according to the first study that tested the validity of *in-vivo* tooth volume determinations from CBCT.^15^

### STRUCTURES INCLUDED IN THE SEGMENTATION PROCESS FOR THE VOLUMETRIC MEASUREMENTS

When considering the structures included in the segmentation, a question arises if the whole tooth should be considered or just the root. Considering only the roots, there is a difficulty of separating the root from the crown. Under normal anatomy, the anatomical crown ends at the cementum-enamel junction (CEJ) level, which is curved. The segmentation tools employed in this study allow an easy straight line or plane segmentation, thanks to the Scalpel function, but not a curved one (Scalpel mode cannot be applied).

If done manually, the curve segmentation would be more challenging and would require a much more significant effort without necessarily increasing the accuracy of the measurement or maybe even decreasing it. This is why the curve segmentation may increase the number of sources of error and subjectivity in the segmentation process. The CEJ is not visible with the employed voxel size, which hinders identifying the limits of the root-crown transition. Besides the technical issues that a curve segmentation generates, the whole tooth segmentation is more precise than the root segmentation alone.^16^

In addition to the previous arguments, the enamel is not expected to change considerably during the average orthodontic treatment time. The attrition wear of human enamel is about 29 microns for molars and about 15 microns for premolars per year,^17^ and the average time of an orthodontic treatment consisting of comprehensive fixed appliances is 24.9 months.^18^ Hence, the amount of enamel wear could be estimated to reach a maximum of around 60 microns for the two years of treatment (0.06 mm). The voxel size used in this study was 0.3 mm. Therefore, the CBCT settings are more commonly employed in private orthodontic offices.^19^ For that reason, any changes detected in the overall tooth volume can be attributed to the root.

For the same technical reasons for which the crown was included, the pulp was included too. The segmentation of the pulp separately from the rest of the tooth structure can be challenging and time-consuming. The inclusion of the pulp chamber in the segmentation label has already been described in a previous in vitro study.^13^ The authors used the “cavity fill” tool in Mimics^®^ to fill the pulp chambers. Besides, even if the pulp volume increases with age^20^ that change would not affect the overall tooth or root volume.

### LIMITATIONS

Several issues may arise when reconstructing 3D volumetric label maps of teeth or roots from CBCT images to assess volumetric pre- and post-treatment changes. These issues are inherent to CBCT imaging and the specific settings during the CBCT acquisition. Some of the reported CBCT issues that may affect the segmentation results are related to the field of view,^11^ ^,^ ^15^ voxel size,^1^ ^,^ ^11^ ^,^ ^21^ partial-volume effect,^13^ ^,^ ^22^ surrounding artifacts,^13^ scatter x-rays,^13^ ^,^ ^19^ ^,^ ^23^ subjectivity in the segmentation process,^11^ ^,^ ^15^ surrounding structures proximity,^15^ ^,^ ^24^ ^,^ ^25^ and movement of the patient during imaging acquisition.^15^ ^,^ ^21^ Thus, the accuracy of the results may present large variability. Another limitation is the length of the process. Given the fact that the segmentation is a very time-consuming procedure, it may not be useful for the busy clinician.

Comparisons to a gold standard should be performed when possible. A comparison with a gold standard like the ones mentioned in the Discussion (laser scan^13^ or water displacement technique^15^ applied to extracted teeth), could not be applied nor used to validate these techniques, since the analyzed teeth were not extracted after treatment.

It should be noted that the same segmentation work, and maybe similar results, could have been achieved with equivalent software, i.e., Avizo^®^. Other software like Dolphin^®^ is not an option, since they do not allow manual segmentation.

## CONCLUSIONS

 The described method seems a reliable way of obtaining tooth volumetric measurements for orthodontic purposes. The reliability level is good to excellent, and the measurement error can vary from 4.16% to 7.11% for intra-rater and inter-rater reliability, respectively.

Nonetheless, any segmentation technique presents limitations due to the inherent characteristics associated with CBCT reconstructions. The measurement error is the reflection of one of those limitations. Oral health professionals need to understand that based on imaging and reconstruction parameters, the volumes depicted may overestimate or underestimate the actual volume, camouflaging real ERR changes that may occur through treatment.

## Figures and Tables

**Figure 1:**
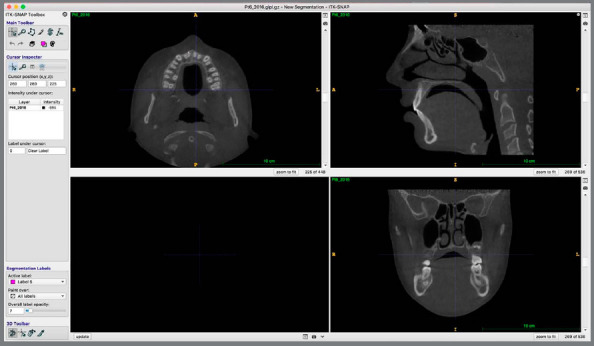
Main image opened, and volume displayed in the three planes.

**Figure 2:**
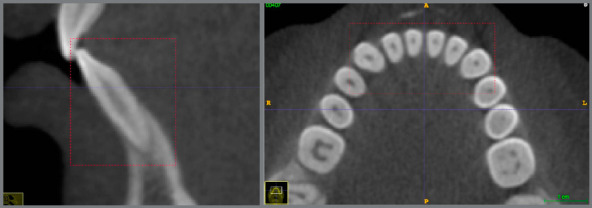
Fitting of the rectangular parallelepiped of the Active Contour Function.

**Figure 3:**
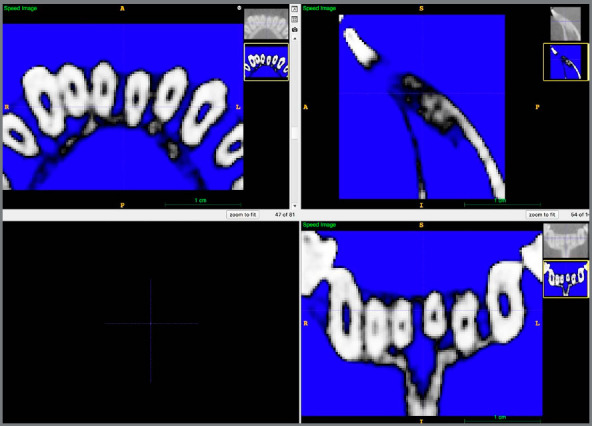
Lower threshold selection.

**Figure 4:**
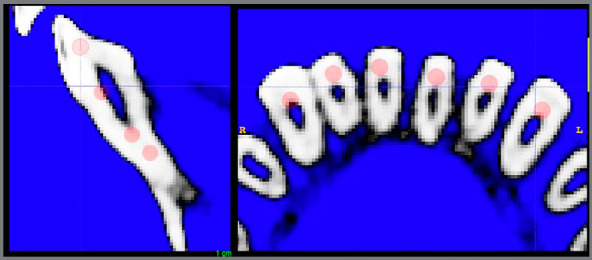
Seeds placed on the teeth.

**Figure 5:**
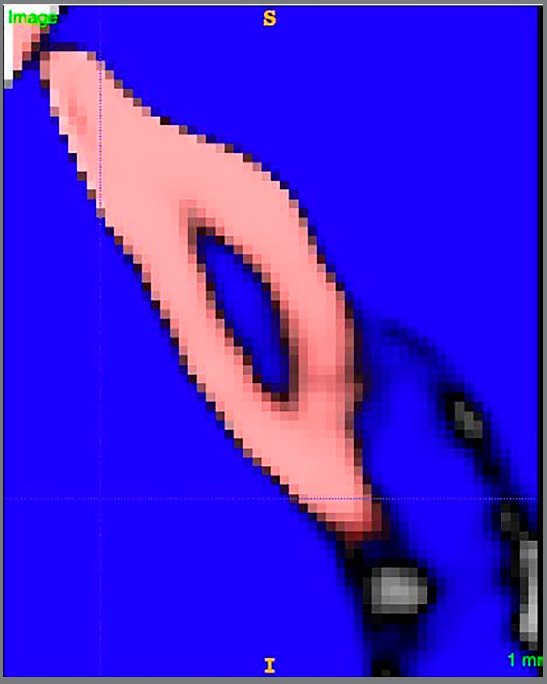
End of the automatic segmentation step.

**Figure 6:**
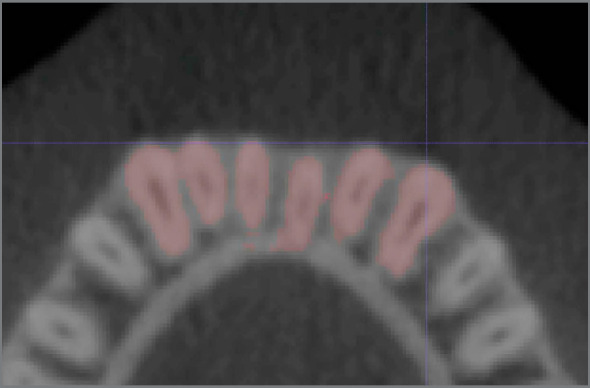
Manual refinement in the axial plane.

**Figure 7:**
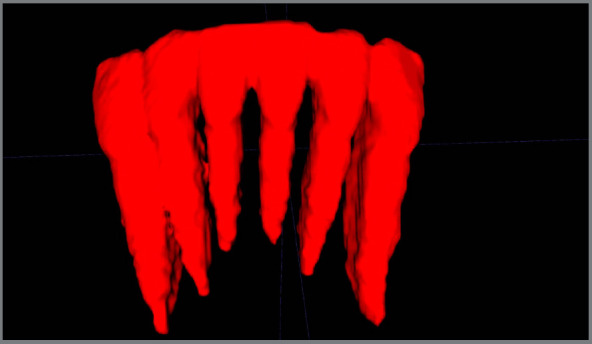
Three-dimensional volume of the teeth previously segmented.

**Figure 8:**
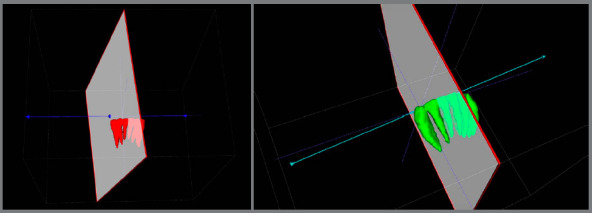
Scalpel Mode being used.

**Figure 9:**
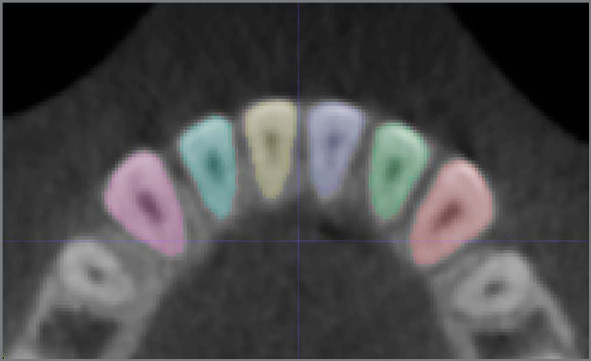
Refinement in the axial plane after using the Scalpel Mode.

**Figure 10, 11:**
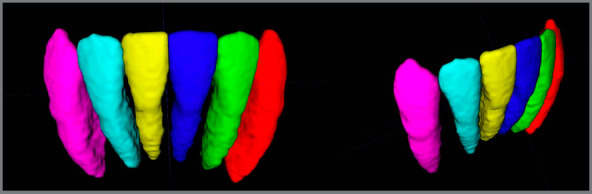
Teeth divided into individual labels with the Scalpel function and after the final axial refinement.

**Figure 12:**
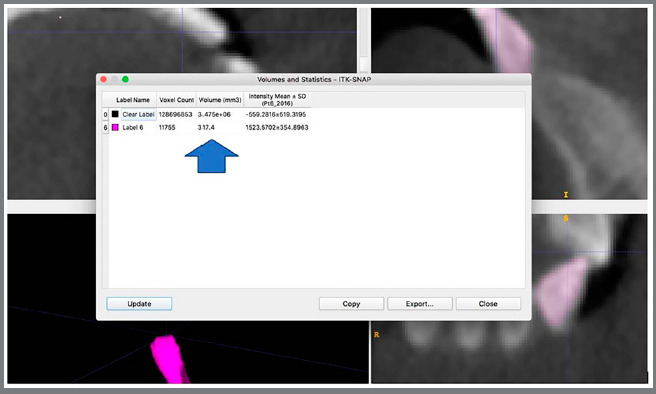
Volume calculation of the segmentation.

## APPENDIX 1 - Step-by-step process of the segmentation for the volumetric measurements

Once ITK-SNAP software^®^ is launched, the display menu from File is used to open a main image, which, in this study, is a CBCT DICOM file. The volume selected is displayed in the three different planes: axial, sagittal and coronal (Fig. 1). Before starting the segmentation process, adjusting the contrast is suggested in order to better depict the image and distinguish the different tissues. The contrast taken to the extremes may make the visualization of the areas of interest difficult.

Using the loop on the Main Toolbar, the anterior sector of the mandible is zoomed into. Once a closer view of the tooth in all the three planes is obtained, the Active Contour Function, aka “Snake”, is selected from the Main Toolbar in order to limit the area of the volume that is necessary for segmentation: lower incisors and canines. Thus, a rectangular parallelepiped is made as fit as possible to the aimed teeth (Fig. 2).

The next step is to push the “Segment 3D” button on the left column. There are several segmentation methods available with ITK-SNAP^®^; the one used for this research is “Threshold”. On the right column, the threshold levels can be changed. The upper threshold is placed to the maximum; whereas the lower threshold is individually adapted for every patient. By arbitrary agreement between the researchers involved in this study, the lower threshold range was considered to be from 500 to 1500. Within that threshold, the operator choses the one that better allows the clearest visualization of the root and crown of the tooth to be measured, without losing tooth structure (Fig. 3). From the author’s experience, switching between the blue and white and the grayscale images helps to gain some insight of the ideal threshold for the specific patient and tooth.

Once the appropriate threshold is selected, the next step consists of adding bubbles or, as called by ITK-SNAP^®^ developers, “seeds”. The seeds are centers of expansion of the segmentation colored label within the previously selected threshold. Their radius was chosen at around one, because it is the size that approximately fits the thinnest areas of the root of a lower incisor and, also, because with smaller seeds the researcher can better visually control the expansion of the segmentation as it occurs. Around four to five seeds per tooth are placed the most equidistant as possible from one to another. The seeds are added close to the center of the teeth, along their long axis and adjacently to the pulp canal and chamber, however, they have to remain within the hard tissue (Fig. 4). These bubbles are going to expand and automatically include the hard tissues of the tooth into the label. Then, the play button is pressed, and the seeds fill the volume of the tooth. In the parameters, the smoothing option has to be set at 0, otherwise, there may be modifications of the surface and volume of the segmented tooth.

Once the tooth is filled, the automatic part of the segmentation is completed, and the researcher can proceed with the manual refinement (Fig. 5).

The manual refinement is necessary because the algorithm used in the automatic segmentation only distinguishes between predetermined thresholds surfaces. Hence, for structures that are really closed in terms of radiopacity, like bone and cementum, the automatic segmentation alone will not be able to clearly differentiate them.

The manual refinement is done thanks to the Paintbrush Mode on the Main Toolbar, but before that, it is convenient to adjust the Overall Label Opacity. By reducing this parameter, the operator can better see the contours of the root and the crown of the segmented teeth, which will allow to differentiate between the alveolar bone and the root. This function does not alter the amount of structure incorporated in the label; it just makes the label more translucent. With the Paintbrush Mode, the colored surface in the label, which is the one that is going to be included in the final volume calculation, can be increased or reduced.

The manual refinement is done in three planes, one at a time, to get the tooth surface and volume closer to the actual anatomy of the tooth (Fig. 6); normally the researcher starts by the axial and sagittal planes, and the coronal plane is rarely used if the previous planes were segmented in detail. The size of the brush can be adapted accordingly to the necessity of adding or removing areas in a more efficient way. The sizes used ranged from two to twelve. In this stage, it is important to also fill the pulp chamber, because the volume calculation is performed including it for the reasons mentioned in the following sections. Overall, this is the most time-consuming step for the researcher.

After revision of the three planes, the update button is pushed, in order to generate the three-dimensional volume of the six teeth together, which, again, helps the researcher to assess if the correct anatomy was delineated (Fig. 7). Any anatomical aberrations, that could be just the result of an improper segmentation process, have to be corrected by going back to the preceding step.

Using the Scalpel Mode (Fig. 8), the teeth can be separated into different labels. This function allows the researcher to create a plane that divides one label into two. It works by tracing a plane in between two teeth and changing the label towards where the arrow points. To segment a specific tooth, more than one cut-plane may be necessary. A final manual refinement in the axial plane, especially at the level of the contact point area, after the use of the Scalpel Function, allows for the most precise tooth structure to be included in the label (Fig. 9). The final three-dimensional model of all the individual labels for all the teeth can be observed in Figures 10 and 11.

Once the segmentation is completed, the software calculates the volume by clicking on Segmentation and then Volume and Statistics (Fig. 12). The volume is given in mm^3^ for each label. Each label corresponds to each of the six measured teeth.
